# Characterizing *KMT2A* Rearrangement in Acute Myeloid Leukemia: A Comprehensive Genomic Study

**DOI:** 10.3390/cancers18010161

**Published:** 2026-01-02

**Authors:** Osama Batayneh, Mahmoudreza Moein, Nour Sabiha Naji, Ansy Patel, Anupa R. Mandava, Alexandra Goodman, Jeffrey S. Ross, Caleb Ho, Chelsea Marcus, Zheng Zhou, Gillian Kupakuwana-Suk, Teresa Gentile, Krishna B. Ghimire

**Affiliations:** 1 Department of Medicine, Division of Hematology/Oncology, SUNY Upstate Medical University, 750 East Adams Street, Syracuse, NY 13210, USA; 2 Department of Medicine, SUNY Upstate Medical University, Syracuse, NY 13210, USA; 3 Novant Health Zimmer Cancer Institute, Wilmington, NC 28401, USA; 4 Foundation Medicine, Cambridge, MA 02141, USA; caho@foundationmedicine.com (C.H.);; 5 Division of Hematology & Hematopoietic Cell Transplantation, City of Hope, Duarte, CA 91010, USA; zzhou@coh.org

**Keywords:** Acute Myeloid Leukemia (AML), *KMT2A* rearrangement (MLL), genomic alterations, molecular study

## Abstract

**Background**: The *KMT2A* (*MLL1*) gene is altered in a variety of hematological malignancies and solid tumors. *KMT2A*-rearranged (*KMT2Ar*) AML represents a distinct subtype associated with poor outcomes and high relapse rate despite initial responsiveness to chemotherapy. **Methods**: A total of 3863 cases of AML peripheral blood samples were analyzed using the FoundationOne Heme combined comprehensive hybrid capture-based DNA and RNA sequencing assay. **Results**: Of the 3863 AML cases, 521 (13.4%) featured genomic alterations (GAs) in the *KMT2A* gene, 99.1% of which were large rearrangements (*KMT2Ar*). A total of 56.9% were males with a median age of 62 years. Of the *KMT2Ar* cases, there were 43.1% *KMT2A* duplications, 52.7% fusions, and 4.2% not otherwise specified rearrangements. A total of 0.9% of the *KMT2A*-altered AML cases were short variant mutations. There were no *KMT2A* (0%) amplifications or deletions. *KMT2Ar* cases were associated with increased GA frequencies in *FLT3* (27.3% vs. 19.8%; *p* = 0.0002), *KRAS* (17.2% vs. 7.8%; *p* < 0.0001) (overall; 1.1% *KRAS* G12C), and *IDH2* (16.0% vs. 10.4%; *p* < 0.0001), while *KMT2A* wild-type AML (*KMT2Awt)* had significantly increased GA frequencies in *RUNX1* (20.7% vs. 15.8%; *p* = 0.0081), *ASXL1* (16.6% vs. 10.5%; *p* = 0.0003), and *TET2* (16.4% vs. 10.1%; *p* = 0.0002), *NPM1* (17.5% vs. 0.2%; *p* < 0.0001), and *TP53* (17.8% vs. 7.9%; *p* < 0.0001). **Conclusions**: *KMT2A* rearrangements are common in AML (13.4% of cases featured *KMT2Ar*). A total of 99.1% of alterations in *KMT2A* are large rearrangements, with fusions being the most commonly observed alteration (52.7% of total rearrangements). No amplifications or deletions were seen. This genomic landscape study highlights significant genomic differences between *KMT2Ar* and *KMT2Awt* AML patients, which may enrich our understanding of the molecular profile and clusters of mutations in AML.

## Simple Summary

*FLT3* and *IDH2* mutations are more commonly seen in *KMT2A*-rearranged acute myeloid leukemia (*KMT2Ar* AML), whereas *NPM1*, *TP53,* and myelodysplasia-related mutations are more commonly seen in *KMT2A* wild-type AML (*KMT2Awt*). This genomic landscape study highlights significant genomic differences between *KMT2A*-arranged and wild-type AML, which may enrich our understanding of the molecular profile and associations between mutations in AML.

## 1. Introduction

Acute myeloid leukemia (AML) encompasses a diverse group of hematologic malignancies, marked by uncontrolled proliferation and clonal expansion of stem and progenitor cells within the bone marrow [1,2]. AML accounts for almost 1.1% of all newly diagnosed cancer cases in the United States, and it is commonly diagnosed at older age, with a median age at diagnosis of 68 years [3,4]. The estimated 5-year overall survival (OS) rate is approximately 32%, although it can vary based on age, with an OS around 50% in younger patients and below 10% in patients over 60 years old [5,6].

The wide range of genomic alterations in AML demonstrates the complexity of this hematologic malignancy [7]. Genomic analysis is a key component in the initial evaluation of AML patients, as it guides treatment decisions through AML subtype classification, prognostication, and risk stratification [8,9]. Comprehensive genomic sequencing of AML conducted by The Cancer Genome Atlas (TCGA), using either whole-genome sequencing or whole-exome sequencing, along with RNA and microRNA sequencing and DNA-methylation analysis, uncovered around 2000 unique somatically mutated genes across a cohort of 200 patients, and at least one driver mutation was identified in nearly all cases of AML [10]. Efforts to characterize the mutational landscape of AML are ongoing and reflected in the initial findings from the Beat AML dataset, which analyzed 672 tumor specimens from 562 patients using whole-exome sequencing, RNA sequencing, and ex vivo drug sensitivity, which identified novel mutational events not previously detected in AML and highlighted predicted drug responses based on specific gene networks [11].

Mutations in epigenetic regulator genes including *KMT2A* are frequently acquired early in AML and are found in the original clonal population, also known as de novo AML [12,13,14]. Such mutations can survive induction chemotherapy, result in clonal expansion in the bone marrow at remission, and eventually lead to leukemic progression and disease relapse [14]. In contrast, mutations in Nucleophosmin (*NPM1)* or other signaling molecules such as *FLT3* and *RAS* usually occur in later leukemogenesis [15].

Lysine-specific N-methyltransferase 2A (*KMT2A*), also known as mixed-lineage leukemia1 (MLL1), is located on chromosome 11q23 and encodes a nuclear protein critical for epigenetic regulation [16]. *KMT2A* belongs to the Trithorax group (TrxG) proteins and facilitates transcriptional activity by catalyzing histone H3 lysine 4 methylation (H3K4me), a key feature of active gene promoters [17,18]. It is frequently involved in chromosomal translocations that generate fusion genes [19].

*KMT2A* is specifically associated with hematologic malignancies due to its crucial role in hematopoietic stem cell (HSC) differentiation [20]. *KMT2Ar* are present in 3–10% of adult AML cases [21]. In general, leukemias with *KMT2A* rearrangements have poor prognosis, with a progression-free survival (PFS) of 30–40% and an overall survival (OS) below 25% [19]. *KMT2A* GAs in AML are heterogeneous in nature and diverse and characterized by chemotherapy resistance and high risk of relapse [22]. A 5-year cumulative incidence of relapse (CIR) of 41.5% was reported in pediatric AML patients with mutated KMT2A, while a higher CIR up to 80% was seen in adult patients [21,23].

*KMT2A* fusion proteins recruit transcription cofactors such as menin, CBX8, TFEb, and DOTL1, which promote transcription elongation [19]. This explains the potential therapeutic effect of menin inhibitors (e.g., Revumenib, Ziftomenib) [24,25] and DOTL1 inhibitors (e.g., Pinometostat) in this adverse disease subtype of AML [21,26,27,28].

The aim of this study is to investigate the major genomic alterations in AML, specifically comparing the different mutations in *KMT2Ar* and *KMT2Awt* AML cases. A comprehensive genomic analysis was performed to characterize key mutations in both groups. Our findings highlight distinct molecular profiles associated with *KMT2Ar*, providing critical insights into the disease mechanisms and the impact of these genomic alterations on prognosis and therapeutic intervention.

## 2. Methods

### 2.1. Study Design

The study protocol received approval from the Western Institutional Review Board (Protocol No. 20152817), including a waiver of informed consent and HIPAA authorization. We performed a retrospective analysis of AML patients who underwent comprehensive genomic profiling (CGP) assays. A total of 3863 peripheral blood samples from patients diagnosed with AML between 2019 and 2024 were included, in which all patients underwent comprehensive genomic profiling of peripheral blood samples with the Foundation One Heme assay, a hybrid capture-based DNA and RNA sequencing platform.

Eligibility criteria included patients who were at least 18 years of age, had a confirmed diagnosis of AML, and had undergone next-generation sequencing (NGS). Genomic data were analyzed for base substitutions, short insertions and deletions, copy number changes and rearrangements, and fusions. Patient age and biological sex were extracted from accompanying pathology reports.

The Foundation One Heme assay includes both DNA and RNA sequencing. The DNAseq component detects the entire coding region of 405 genes and selects intronic regions in 31 genes known to be clinically and biologically relevant in cancer. The RNAseq component is focused on 265 genes recurrently rearranged in cancer. This assay is validated to a high accuracy, achieved through high and uniform coverage, with an average median exon depth of 500× (DNA) and average on-target distinct pairs of ~3 M (RNA). The tumor mutational burden (TMB) is defined as the total number of mutations found in the DNA of cancer cells and is reported as the number of mutations seen in a section of DNA per megabase (mut/Mb) (a TMB of 10 mut/Mb or greater was referred to as TMB-high) [29]. Microsatellite stability (MSS) status was determined on at least 1500 loci [30,31]. The Catalogue of Somatic Mutations in Cancer (COSMIC) was used in order to reflect the underlying mechanisms of mutational processes in each case [32].

**Figure 1:**
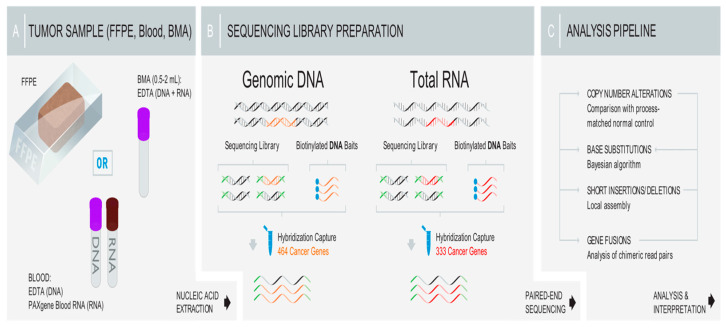
Workflow of the Foundation Heme One assay, illustrating a comprehensive combined DNA and RNA genomic analysis in clinical specimens and the coverage distribution of all exons.

The FoundationOne Heme assay was utilized for comprehensive genomic profiling as previously described [33], and the sequencing workflow is demonstrated in Figure 1.

Patients were categorized into two cohorts for comparative analysis based on their genomic profiling results: patients diagnosed with *KMT2A* rearrangements (*KMT2Ar*) and those without *KMT2A* rearrangements, also referred to as *KMT2A* wild-type AML (*KMT2Awt*).

### 2.2. Outcome Definitions

The primary aim of this study was to characterize the genomic landscape of AML, with emphasis on comparing key genetic alterations between the *KMT2Ar* and *KMT2Awt* AML cases. Secondary objectives included assessing the distribution of TMB, and MSS status in these cohorts, as well as examining potential associations between genomic profiles and available clinical characteristics such as patient age, gender, laboratory markers, and treatment response.

The analysis included clinical endpoints and variables such as median age at diagnosis, prevalence of specific genomic alterations, TMB classification (above 10 mutations/Mb and above 20 mutations/Mb), MSS status, and mutational signature patterns referenced from COSMIC.

### 2.3. Statistical Analyses

The median age of the study population was calculated. Comparisons between the *KMT2A*-rearranged (*KMT2Ar*) and *KMT2A* non-rearranged (*KMT2Awt*) groups were performed. Univariate analyses of continuous variables were conducted using the t-test, while categorical variables were assessed using the chi-square test or Fisher’s exact test, as appropriate. Continuous data are presented as means with standard deviations (±SD), and categorical data are reported as counts and percentages. To account for multiple comparisons and control the false discovery rate, Fisher’s exact test results were adjusted using the Benjamini–Hochberg procedure, which ranks individual *p*-values and applies a progressively stricter significance threshold as the number of tests increases. A two-sided *p*-value < 0.05 was considered statistically significant.

## 3. Results

A total of 3863 AML cases were included in the analysis. Based on *KMT2A* genomic alterations, cases were stratified into two subgroups: 521 (13.5%) cases of *KMT2A*-rearranged, in which 99.1% of the GAs were large rearrangements, and 3342 (86.5%) cases of *KMT2A* non-rearranged (wild-type).

There were no significant differences between the *KMT2Ar* and *KMT2Awt* cohorts with respect to sex or age (mean age: 52.8 vs. 53.7 years; *p* = NS, or median age: 61 vs. 62 years; *p* = NS) (Table 1).

Within the *KMT2Ar* subgroup, alteration types were distributed as follows: 43.1% duplications, 52.7% fusions, and 4.2% rearrangements not otherwise specified (NOS). Short variant mutations accounted only for 0.9% of *KMT2A*-altered cases, and no *KMT2A* amplifications or deletions were observed.

**Figure 2:**
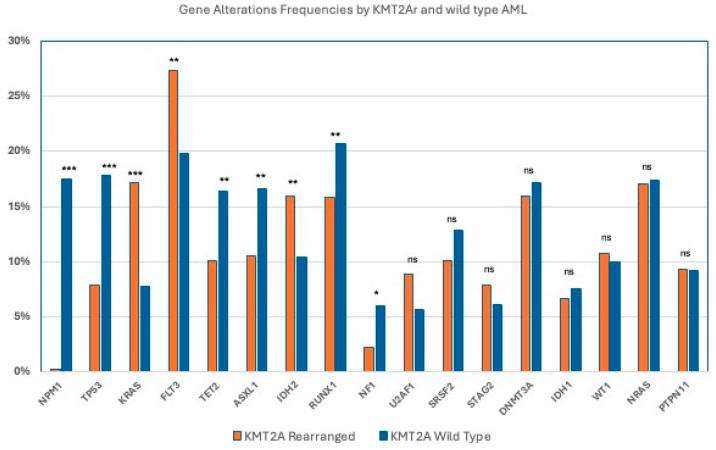
Gene alteration frequencies between *KMT2A*-rearranged and *KMT2A* wild-type AML Cases. *: *p* < 0.05. **: *p* < 0.001. ***: *p* < 0.0001. ns: not statistically significant.

When comparing the genomic landscape between *KMT2Ar* and *KMT2Awt* AML cases, several significant differences were observed and are demonstrated in Table 2 and Figure 2. The *KMT2Ar* cases demonstrated significantly higher frequencies of GAs in *FLT3* (27.3% vs. 19.8%; *p* = 0.0001), *KRAS* (17.2% vs. 7.8%; *p* < 0.0001), and *IDH2* (16.0% vs. 10.4%; *p* < 0.0001).

In contrast, the *KMT2Awt* cases demonstrated significantly higher frequencies of GAs observed in *RUNX1* (20.7% vs. 15.8%; *p* = 0.0081), *ASXL1* (16.6% vs. 10.5%; *p* = 0.0003), *TET2* (16.4% vs. 10.1%; *p* = 0.0002), *NPM1* (17.5% vs. 0.2%; *p* = <0.0001), and *NF1* (6% vs. 2.2%; *p* = 0.001). *TP53* mutations were significantly increased in *KMT2Awt* (17.8% vs. 7.9%; *p* = <0.0001)

No significant differences were observed between the two groups in the prevalence of GAs in *NRAS* (17.4% vs. 17%), *DNMT3A* (16.0% vs. 17.2%), *WT1* (10.7% vs. 10%), *SRSF2* (10.1% vs. 12.8%; *p* = NS), *PTPN11* (9.3% vs. 9.2%; *p* = NS), *U2AF1* (8.9% vs. 5.7%; *p* = NS), *STAG2* (7.9% vs. 6.1%; *p* = NS), or *IDH1* (6.7% vs. 7.6%; *p* = NS).

With respect to mutational burden, the median tumor mutational burden (TMB) was similar between the *KMT2Ar* and *KMT2Awt* cohorts at 0.8 mutations/Mb, with only 0.2% of cases in either group exhibiting a TMB greater than 10 mutations/Mb. Furthermore, all cases (100%) in both groups were microsatellite-stable (MSS).

## 4. Discussion

Recently, AML with *KMT2Ar* has been widely studied, as it is highly characterized by adverse outcomes due to treatment failure, high relapse rate, and early mortality [34]. The t(9;11) (p21.3;q23.3) translocation resulting in the *KMT2A*-*MLLT3* fusion is the most frequent *KMT2A* rearrangement seen in adults with AML, although over 80 different fusion partners have been identified [35].

*KMT2Ar* results in fusion proteins that drive leukemia by activating HOX genes and their cofactor Meis1, which are both critical for leukemogenesis [36]. Menin serves as an essential scaffolding protein that facilitates the binding of *KMT2A* complex to HOX gene promoters, playing an important role in pathogenesis [37]. The disruption of the menin-*KMT2A* interaction can restore normal gene expression, promote differentiation, and exert an antileukemic effect. Similarly, AML with NPM1 mutations depends on the menin–*KMT2A* interaction, and thus the efficacy of menin inhibitors is studied in AML with *NPM1* mutation and *KMT2A* rearrangement [38].

Despite the use of intensive chemotherapy and hematopoietic stem cell transplants (HSCTs), patients with *KMT2Ar* AML still have unfavorable prognosis and show limited responses to treatment [39]. Additionally, therapy-related *KMT2Ar* AML has worse OS compared to de novo *KMT2Ra* AML [23]. Of the chemotherapeutic agents linked to therapy-related AML, topoisomerase II inhibitors (such as daunorubicin) are closely linked to *KMT2Ar* AML and typically lead to disease onset after a short latency period [40].

Menin inhibitors are emerging therapeutic agents under clinical investigation in *NPM1*-mutated and *KMT2Ar* AML, with a promising effect on the transcriptional network driving leukemogenesis [24]. Revumenib is a menin inhibitor that received its first FDA approval in the United States in November 2024 for the treatment of relapsed/refractory *KMT2Ar* AML [28,41,42]. Ongoing clinical trials are evaluating integrating menin inhibitors into upfront standard induction/consolidation therapy. [43] Other menin inhibitors such as Ziftomenib [25] and Bleximenib [44] are being studied in addition to the possible combination therapies with venetoclax, hypomethylating agents, PARP inhibitors, all-trans-retinoic acid (ATRA), and other common treatment options in AML [45].

In our study, the percentage of *KMT2Ar* in AML is 13.5%, which is slightly higher than the reported rate of 3–10% in the literature [21]. This could be attributed to the retrospective study design which included samples at diagnosis and variable times throughout the course of treatment. There was no significant difference based on sex between *KMT2Ar* and *KMT2Awt* groups, which aligns with the published data [46,47]. Similarly, there was no significant difference based on age between the two groups. Interestingly, a large proportion of our patient samples (43.1%) had duplications as GAs in *KMT2A*, which is higher than the reported cases (3–11%) [48,49,50,51]; this could be potentially explained by our assay using combined DNA and RNA sequencing in comparison to other studies utilizing DNA-based testing only. Short variant mutations are very rare (0.9%) in *KMT2A* AML compared to rearrangements and duplications, and the latter aligns with the available data [52].

Importantly, genomic alterations, which serve as potential targets for treatment, show higher frequency in *KMT2Ar* cases compared to *KMT2wt*. These include GAs in *FLT3* and *IDH2*. Additionally, GAs in *KRAS*, including *KRAS G12C* mutations, were more frequently seen in *KMT2Ar* AML, which has more clinical implications in solid malignancies like non-small-cell lung cancer. [53] *KMT2Ar* is the primary initiating event and key driver in *KMT2Ar* AML, while the effect of additional mutations on prognosis and clinical outcomes is under investigation [54,55]. In a study by Issa et al., *RAS* and *FLT3* mutations in *KMT2Ar* AML did not have a significant implication on prognosis except when ≥2 mutations were present [23]. However, a recent study by Wu et al. showed that *KMT2Ar* AML patients harboring *KRAS* mutations had significantly worse 2-year OS and higher 2-year cumulative incidence of relapse (CIR) compared to those with wild-type *KRAS*, while *NRAS* and *FLT3* mutations were not significantly associated with differences in OS or CIR [56]. Studies suggest that *KMT2A* rearrangement creates a cellular environment favorable for the acquisition and maintenance of KRAS mutations, which have a synergistic leukemogenesis effect [57,58]. In preclinical studies, it has been demonstrated that FLT ITD and NRAS mutations accelerate *KMT2A* leukemia onset, possibly by providing stimulatory factors [58,59].

On the other hand, we found a significantly lower frequency of GAs in *RUNX1*, *ASXL1*, *TET2* and *NF1* in *KMT2Ar* compared with *KMT2Awt* cases. Mutations in genes like ASXL1 and TET2 are often mutated in the elderly and are associated with clonal hematopoietic expansion, with a high risk of developing hematologic malignancies [60].

There was also no significant difference in the prevalence of GAs in NRAS, DNMT3A, WT1, SRSF2, PTPN11, U2AF1, STAG2, or IDH1 between both groups. Other studies also demonstrated a low prevalence of these mutations in *KMT2Ar* AML [23,55]. *NPM1* mutation was exclusively observed in *KMT2Awt* (17.5% vs. 0.2%; *p* < 0.0001), suggesting that in patients with *KMT2Ar* AML, the chances of identifying *NPM1* are extremely low, at <0.5%.

*TP53* mutations represent a significant challenge in AML and MDS due to their resistance and high rates of relapse with conventional chemotherapy [61,62,63,64]. The rate of *TP53* mutations in our study was significantly higher in patients with *KMT2Awt* compared to *KMT2Ar* AML (17.8% vs. 7.9%; *p* < 0.0001), which correlates with the literature [63].

Regarding mutational burden, we reported a similar TMB of 0.8 mutations/Mb between the *KMT2Ar* and *KMT2Awt* cohorts, with only 0.2% of cases in either group exhibiting a TMB greater than 10 mutations/Mb. All cases in both groups were microsatellite-stable (MSS). The median TMB identified in the literature is equally low, with 0.5 mutations per patient in *KMT2Ar* and 2 mutations per patient in KMT2Anr [23]. Unlike solid tumors, in which TMB and/or MSS status have been validated as biomarkers and have clinical implications regarding responsiveness to immunotherapy [65], testing in AML patients is not routinely performed, and there is no available data about MSS and TMB testing in *KMT2Ar* AML.

The main limitation of this study was the lack of data on clinical outcomes in this studied population.

## 5. Conclusions

*KMT2A* rearrangements are common in AML (13.4% of cases featured *KMT2Ar*). A total of 99.1% of alterations in *KMT2A* are large rearrangements, with fusions being the most commonly observed alteration (52.7% of total rearrangements). No amplifications or deletions were seen. *FLT3* and *IDH2* mutations are more commonly seen in *KMT2Ar* AML, whereas *NPM1*, *TP53,* and myelodysplasia-related mutations are more commonly seen in *KMT2Awt*. This genomic landscape study highlights significant genomic differences between *KMT2Ar* and *KMT2Awt* AML patients, which may enrich our understanding of the molecular profile and clusters of mutations in AML.

## Figures and Tables

**Table 1:** Baseline characteristics comparison between *KMT2A*-rearranged vs. *KMT2A* wild-type AML patients. ns: not statistically significant.

Patient Characteristics	Total Cohort (*n* = 3863)		*p*-Value
	*KMT2A* Rearranged (*n* = 521)	*KMT2A* Wild Type (*n* = 3342)	
Sex			ns
Male	301 (57.5%)	1875 (56.1%)	
Female	220 (42.5%)	1467 (43.9%)	
Age (median, range)	61 (1–89)	62 (1–89)	ns
Age (mean)	52.5	53.7	ns

**Table 2:** Genomic analysis comparison between *KMT2A*-rearranged AML vs. *KMT2A* wild-type AML patients. ns: not statistically significant.

	Total Cohort (*n* = 3863)		*p*-Value
	*KMT2A* Rearranged (*n* = 521)	*KMT2A* Wild Type (*n* = 3342)	
Pathogenic genomic alterations			
*FLT3*	27.3%	19.8%	0.0002
*NRAS*	17%	17.4%	ns
*KRAS*	17.2%	7.8%	<0.0001
*TP53*	7.9%	17.8%	<0.0001
*NPM1*	0.2%	17.5%	<0.0001
*DNMT3A*	16%	17.2%	ns
*IDH2*	16%	10.4%	0.0004
*RUNX1*	15.8%	20.7%	0.0081
*WT1*	10.7%	10.0%	ns
*ASXL1*	10.5%	16.6%	0.0003
*TET2*	10.1%	16.4%	0.0002
*SRSF2*	10.1%	12.8%	ns
*PTPN11*	9.3%	9.2%	ns
*U2AF1*	8.9%	5.7%	ns
*STAG2*	7.9%	6.1%	ns
*IDH1*	6.7%	7.6%	ns
*NF1*	2.2%	6.0%	0.001
Microsatellite instability (MSI)			
MSI-high	0	0	1
Tumor mutational burden (TMB)			
Median TMB (mut/Mb)	0.8	0.8	ns
Mean TMB (mut/Mb)	1.5	1.4	ns
TMB ≥ 10 mut/Mb	0.2%	0.2%	ns
TMB ≥ 20 mut/Mb	0.2%	0.1%	ns

## Data Availability

The raw data supporting the conclusions of this article will be made available by the authors on request.
